# Evaluation of *Bacillus licheniformis*-Fermented Feed Additive as an Antibiotic Substitute: Effect on the Growth Performance, Diarrhea Incidence, and Cecal Microbiota in Weaning Piglets

**DOI:** 10.3390/ani10091649

**Published:** 2020-09-14

**Authors:** Kuei-Hung Lin, Yu-Hsiang Yu

**Affiliations:** Department of Biotechnology and Animal Science, National Ilan University, Yilan 26047, Taiwan; kueihunglin0101@gmail.com

**Keywords:** antibiotic, *Bacillus licheniformis*, diarrhea, microbiota, piglet

## Abstract

**Simple Summary:**

Many countries have already restricted the use of antibiotics in preventing diarrhea and improving the growth of weaning piglets. Therefore, exploring alternatives to antibiotics is an urgent unmet need. Bacillus licheniformis-fermented feed additive (BLF)-containing probiotics and antimicrobial substances can diminish the diarrhea incidence of weaning piglets. However, limited information is available in terms of the parallel supplementation of antibiotics and BLF in the diet of weaning piglets. For practical application, this information is important to assess whether BLF can be used as an antibiotic substitute. In this study, the effects of BLF or in combination with bacitracin (antibiotics) on weaning piglets were evaluated. The results showed that the half replacement of bacitracin with BLF leads to positive effects on the alleviation of diarrhea incidence and modification of cecal microbiota in weaning piglets.

**Abstract:**

This study investigated the potential of a *Bacillus licheniformis*-fermented feed additive (BLF) as an antibiotic substitute in weaning piglets. Ninety-six crossbred piglets were randomly allotted into four treatments with three replicate pens per treatment and eight pigs per pen. Piglets were fed diets as follows: a basal diet as control, a basal diet supplemented with bacitracin (30 mg/kg of bacitracin methylene disalicylate), a basal diet supplemented with BLF (1 g/kg of the *Bacillus licheniformis*-fermented feed additive), and a basal diet supplemented with bacitracin and BLF (15 mg/kg of bacitracin methylene disalicylate and 0.5 g/kg of the *Bacillus licheniformis*-fermented feed additive). The results showed that replacing all or half the bacitracin with BLF both reduced the incidence of diarrhea in weaning piglets from day 1 to 14. Principal coordinates analysis and a species abundance heat map showed that distinct clusters were formed between groups. Replacing all the bacitracin with BLF reduced bacterial evenness in the cecal digesta of weaning piglets, while the inhibitory effect on bacterial evenness was reversed in the group treated with bacitracin in combination with BLF. These results indicated that the half replacement of bacitracin with BLF was able to decrease the incidence of diarrhea and modify cecal microbiota composition in weaning piglets, suggesting that a *Bacillus licheniformis*-fermented feed additive has good potential as a suitable alternative to antibiotics use in the swine industry.

## 1. Introduction

Intestinal diseases and diarrhea in weaning piglets caused by environmental stress and conditioned pathogens are major economic problems in the swine industry worldwide [1]. Intestinal microbiota disturbances after weaning are associated with a high incidence of diarrhea and lead to poor growth rate in piglets [2]. The use of antibiotics as growth promoters has been effective at reducing post-weaning diarrhea and promoting immunomodulatory and anti-inflammatory responses, thereby improving the growth of weaning piglets [3,4]. However, multi-drug resistant pathogens and antibiotic residues in animal products are becoming a global health concern due to the overuse of antibiotics in animal production. Many countries have banned antibiotic growth promoters for animal growth promotion. Hence, finding acceptable alternatives to antibiotics in the prevention of diarrhea in weaning piglets is important.

Several alternatives to antibiotics have been developed in recent years, among which probiotics have been prominent [5,6,7]. It has been reported that in weaning piglets, probiotic supplementation in diets can have a similar effect to antibiotics [8]. The prevention of post-weaning diarrhea by the application of probiotics or probiotic-derived functional metabolites has also been investigated in weaning piglets [9,10,11]. Among the probiotic strains that have been studied, *Bacillus licheniformis* has been reported to have a strong ability to produce digestive enzymes and antimicrobial peptides that enhance nutrient utilization and inhibit pathogen growth [12,13,14]. Furthermore, the combined use of *B. licheniformis* and *Bacillus subtilis* have been found to decrease the morbidity and mortality rates, ameliorate enteritis, and improve the growth performance of weaning piglets [15,16]. *B. licheniformis* supplementation has also been reported to improve the incidence of diarrhea in weaning piglets [17]. Our recent study also demonstrated that a *B. licheniformis*-fermented feed additive (BLF) has a beneficial effect on the alleviation of the diarrhea incidence of weaning piglets [11]. More importantly, bacterial communities in the feces were clearly distinct in the weaning piglets treated with antibiotics and those treated with BLF [11].

The combination of antibiotics and probiotics, as well as comparisons of these feed additives, have been evaluated in broilers and piglets [18,19,20]. The half replacement of antibiotics with *Bacillus amyloliquefaciens* has been found to improve the growth performance, antioxidant activity, and digestive enzyme activity in weaning piglets [18,19]. However, to the best of our knowledge, limited information is available in terms of the parallel supplementation of bacitracin and BLF in the diet of weaning piglets. It is important to comparatively evaluate the beneficial effects of bacitracin and BLF for practical application. Therefore, this study aimed to investigate the effects of BLF on the growth performance, diarrhea incidence, and cecal microbiota of weaning piglets as a substitute for antibiotics.

## 2. Materials and Methods

### 2.1. BLF and Antibiotics

*B. licheniformis* (American Type Culture Collection 12713) was obtained from the Food Industry Research and Development Institute (Hsinchu, Taiwan). Protocols for BLF preparation in this study were conducted as described previously [21]. Briefly, sterile fermentation substrates were inoculated with the inoculum of *B. licheniformis* and incubated at 30 °C for 6 days. Fermented feeds were dried at 50 °C and then homogenized through mechanical agitation. The *B. licheniformis* spore and *B. licheniformis*-derived antimicrobial peptide (surfactin) quantities in BLF were 5 × 10^11^ CFU/g and 10 mg/g, respectively. Bacitracin methylene disalicylate was obtained from Nice Garden Industrial (Taipei, Taiwan).

### 2.2. Animals and Diets

All the procedures used in this experiment were approved by the Institutional Animal Care and Use Committee of National Ilan University (IACUC, protocol number 109-4). Ninety-six crossbred castrated male and female piglets ((Landrace × Yorkshire) × Duroc) at 28 days old with an average initial body weight of 9.31 ± 0.638 kg were randomly divided into four groups in a completely randomized design. Each group had three replicates with eight piglets per replicate. The dietary treatments comprised a basal diet as control (C), a basal diet supplemented with 30 mg/kg of bacitracin (bacitracin methylene disalicylate) (A), a basal diet supplemented with 1.0 g/kg of BLF (5 × 10^8^ CFU/kg of feed) (BLF), and a basal diet supplemented with 15 mg/kg of bacitracin (bacitracin methylene disalicylate) and 0.5 g/kg of BLF (2.5 × 10^8^ CFU/kg of feed) (AF). Diets were formulated to meet or exceed the nutrient requirement recommendation by National Research Council (Nutrient Requirements for Swine, 2012, Table 1). In BLF-treated groups, the soybean meal in the basal diet was equally replaced with BLF. Food and water were offered ad libitum throughout the experimental period. The experimental period was 28 days. All piglets were adapted to the pens and the basal diets for 7 days before starting the experiment. The piglets were housed in pens with slatted metal floors (2.5 m × 4.0 m). The room temperature was 30 °C at the arrival of the piglets and was then gradually decreased and kept at 24 °C until the end of the experiment. A 10 light: 14 dark photoperiod was applied throughout the experiment. The growth performance parameters (average body weight, average daily weight gain, average daily feed intake, and feed conversion ratio) were measured every week.

### 2.3. Analysis of Diarrhea Incidence

The consistency of the fresh excreta was evaluated daily according to 4 levels: 0, solid; 1, semi-solid; 2, semi-liquid; and 3, liquid. Levels 0 and 1 were considered normal, while levels 2 and 3 were considered diarrhea. The incidence of diarrhea in each pen was recorded daily and calculated as described previously [10]

### 2.4. Small Intestinal Morphology

Two piglets per replicate pen were randomly chosen (based on the average weight of each replicate) at the end of the experiment (day 28) and euthanized by electrical stunning followed by exsanguination. Three replicates (6 piglets per treatment, n = 3) were used for the examination of intestinal histology. Two-centimeter-long segments of the duodenum, jejunum, and ileum were taken from the middle of each part and immediately fixed in 10% neutral buffered formalin (Origin Pure Biosci and Tech, Taipei City, Taiwan). All specimens were sectioned at a thickness of 5 μm (3 cross-sections from each sample) on a rotary microtome (Thermo Scientific, Waltham, MA, USA), followed by staining with hematoxylin and eosin. An image analyzer system composed of an Olympus CKX41 microscope (Olympus Corporation, Tokyo, Japan) and the Visiopharm image software (Hørsholm, Denmark) was used for estimating intestinal morphology. The villus length, crypt depth, and villus-to-crypt ratio of each segment were measured randomly on 30 villi in one piglet.

### 2.5. 16S Ribosomal RNA Sequencing and Analysis

Two piglets per replicate (6 piglets per treatment, n = 3) were randomly chosen (based on the average weight of each replicate) at the end of the experiment (day 28) and euthanized by electrical stunning followed by exsanguination. The cecal digesta from 2 piglets were freshly collected and then pooled for cecal microbiota analysis. The total genomic DNA from cecal digesta was extracted using a QIAamp DNA Microbiome kit (QIAGEN, Germantown, MD, USA). Purified genomic DNA was quantified on a Quibt 2.0 Fluorometer (Thermo Scientific, Waltham, MA, USA). The PCR amplification of the variable regions 3 and 4 of the 16S rRNA gene was conducted using region-specific primers (341F-805R). The PCR products were purified by agarose gel electrophoresis, and the QIAquick Gel Extraction kit (QIAGEN, Germantown, MD, USA) was used for the recovery of DNA from the gels. All sequencing libraries were generated using TruSeq Nano DNA Library Prep kits (Illumina, San Diego, CA, USA), and the libraries were quantified on the Qubit 2.0 Fluorometer (Thermo Scientific, Waltham, MA, USA) and Agilent 2100 Bioanalyzer (Agilent Technologies, Santa Clara, CA, USA). The libraries were subjected to paired-end sequencing (2  ×  300 bp) by the Illumina MiSeq platform. Amplicon sequences were clustered into operational taxonomic units (OTUs) using a similarity threshold of 97% in the UCHIME software (version 4.2) and the mothur software (version 1.39.5). The obtained sequences were aligned to the Genomes Online Database (gold.jgi.doe.gov) to determine the OTUs’ phylogeny. Low abundance OTUs (<0.005% of total reads) were removed. A Venn diagram was constructed to present the number of unique and shared OTUs (version 1.6.17). Taxonomic assignments and alpha diversity analysis were made using a naïve Bayesian classification method (rdp.cme.msu.edu) and the QIIME 2 software (version 2017.4, GitHub, San Francisco, CA, USA), respectively. The principal component analysis (PCA), principal coordinate analysis (PCoA), and beta diversity were calculated using the weighted and unweighted UniFrac metric (a distance metric used for comparing bacterial communities) and performed in the QIIME 2 software. The R corrplot package (v.0.84, GitHub, San Francisco, CA, USA) was used to visualize the correlation matrices.

### 2.6. Statistical Analysis

Data were analyzed by a one-way ANOVA through the general linear model procedure of statistical analysis system (SAS) (version 9.4, 2012; SAS Institute, Cary, NC, USA). Replicates were used as the experimental unit. Means were compared using Tukey’s honestly significant difference test. A *p* value between 0.05 and 0.1 was considered a trend, and a *p* value of less than 0.05 was statistically significant. The PCA, PCoA, and beta diversity were performed in the QIIME software using UniFrac distances coupled with standard multivariate statistics. *p* values were adjusted for multiple comparisons using the Benjamini–Hochberg false discovery rate method. The correlations between cecal microbiota, diarrhea incidence, and growth performance were analyzed using Pearson’s correlation coefficient.

## 3. Results

### 3.1. Effect of BLF as an Antibiotics Substitute on Growth Performance, Diarrhea Incidence, and Small Intestine Morphology

All piglets were healthy over the experimental period. Though the changes in body weight between groups were not statistically significant, a trend of increased body weight was observed with the supplementation of BLF in combination with bacitracin (27.24 kg) compared with the control group (26.40 kg) (*p* = 0.066). Though it did not reach statistical significance, the half replacement of bacitracin with BLF caused a trend in improving the average daily weight gain in weaning piglets at 1–28 days (0.63 kg/d/head) compared with the control group (0.601 kg/d/head) (*p* = 0.061). There were no significant differences between groups in average daily feed intake and feed conversion ratio over the experimental period. The incidence of diarrhea in the group treated with bacitracin was significantly decreased compared with the control group at 1–14 days (*p* < 0.05) (Table 2). Similar to bacitracin, replacing all the bacitracin or the half replacement of bacitracin with BLF also reduced the diarrhea incidence in weaning piglets compared with the control group at 1–14 days (*p* < 0.05) (Table 2). No significant difference was observed in the incidence of diarrhea between the group treated with bacitracin alone, BLF alone, and BLF in combination with bacitracin (Table 2). There were no significant differences between groups in diarrhea incidence at 15–28 days (Table 2). Though the changes in diarrhea incidence between groups were not statistically significant at 1–28 days, a trend of decreased diarrhea incidence in weaning piglets was observed with the supplementation of bacitracin compared with the control group (*p* = 0.058) (Table 2).

The villus length was longer in the small intestine of the duodenum in the antibiotics alone group compared with the control group (*p* < 0.05) (Table 3). Though it did not reach statistical significance, replacing all the bacitracin with BLF caused a trend in decreasing crypt depth in the small intestine of the jejunum (*p* = 0.062) (Table 3). Similar to bacitracin, replacing all bacitracin with BLF increased the ratio of villus length to crypt depth in the small intestine of the ileum compared with the control group (*p* < 0.05) (Table 3).

### 3.2. Effect of BLF as an Antibiotics Substitute on the Cecal Bacterial Microbiota

**Figure 1:**
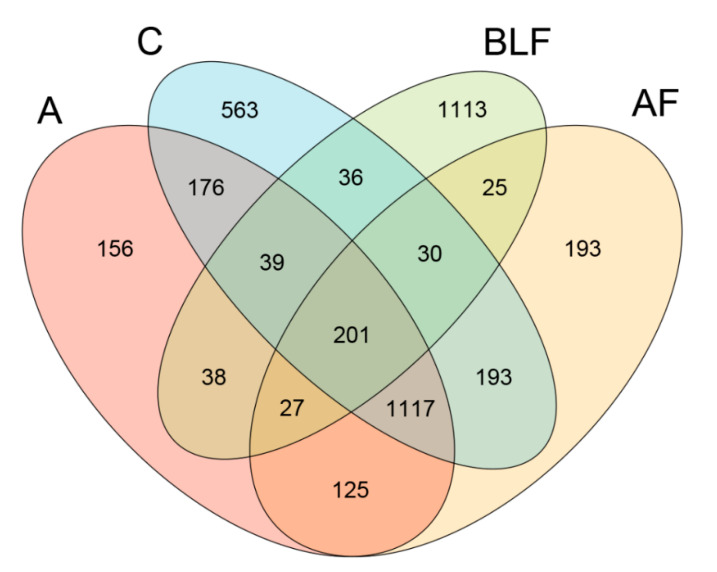
Venn diagram showing the number of OTUs shared and unique between groups. The overlapping regions between the ellipses illustrate the OTUs that were shared between the C, A, BLF, and AF groups (n = 3). The value of each region represents the number of OTUs corresponding to the region.

**Figure 2:**
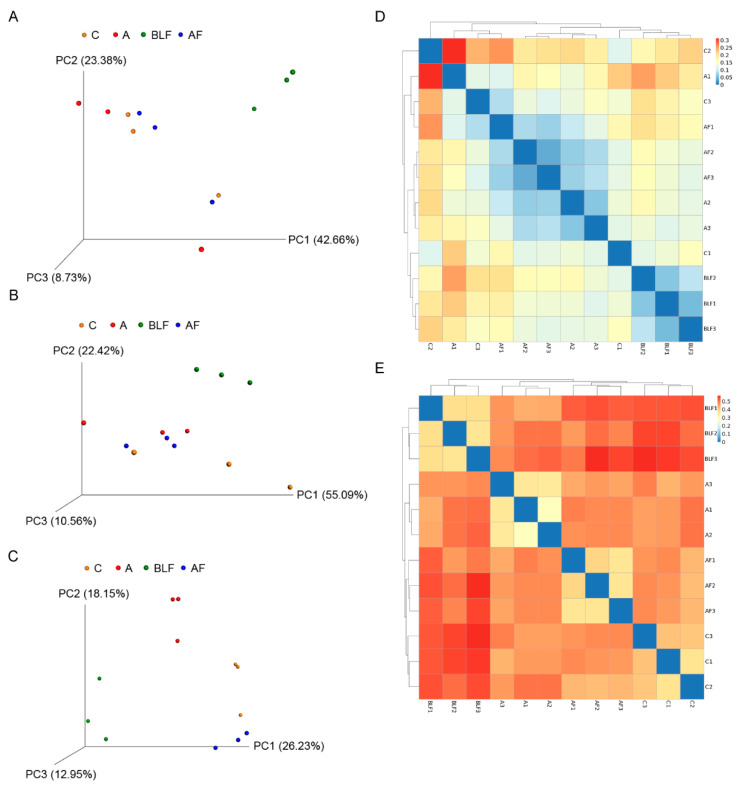
Advanced analysis of the bacterial communities of cecal digesta. (**A**) Principal component analysis plots of the cecal digesta of the C, A, BLF, and AF groups (n = 3). Principal coordinate analysis plots of quantitative traits (weighted UniFrac distances) (**B**) and qualitative traits (unweighted UniFrac distances) (**C**) of the cecal bacterial communities from the C, A, BLF, and AF groups (n = 3). (**D**) The beta diversity index of the cecal digesta from the C, A, BLF, and AF groups based on weighted UniFrac distances (n = 3). (**E**) The beta diversity index of the cecal digesta from the C, A, BLF, and AF groups based on unweighted UniFrac distances (n = 3).

After the quality-based trimming of raw data, the averages of high-quality reads from the cecal digesta of weaning piglets fed only a basal diet, bacitracin, BLF, or BLF in combination with bacitracin were 84,566, 97,807, 87,001, and 89,312, respectively. The average OTUs in the four aforementioned groups were 20,139, 23,336, 21,042, and 21,133, respectively (Table 4). Alpha diversity results showed that no significant difference was observed in the cecal species richness (Chao1 and Fisher alpha) between groups (Table 4). In contrast, one of the cecal species evenness estimators (Shannon) was decreased in the group treated with BLF alone compared with the control group (*p* < 0.05) (Table 4). The Venn diagram indicated that 201 OTUs (core overlap) were shared by four of the plotted groups (Figure 1). The numbers of unique OTUs found in the four aforementioned groups were 563, 156, 1113, and 193, respectively. On one hand, 176 OTUs were found in both the control group and the group treated with bacitracin alone, and 36 OTUs were found in both the control group and the group treated with BLF alone. In contrast, 193 OTUs were found in both the control group and the group treated with BLF in combination with bacitracin. The PCA indicated statistically significant discrimination among the groups (Figure 2a). The PCoA of quantitative traits and qualitative traits revealed that the microbiota of cecal samples among the groups were clearly separated (Figure 2b,c). The beta diversity analysis based on quantitative traits and qualitative traits also revealed that the cecal microbiota among the groups was clearly differentiated (Figure 2d,e). As found through quantitative analysis, replacing all bacitracin with BLF exhibited a unique cecal bacterial structure. However, the cecal bacterial structure between groups was clearly separated based on qualitative analysis.

### 3.3. Effects of BLF as an Antibiotics Substitute on the Cecal Bacterial Taxonomic Composition

**Figure 3:**
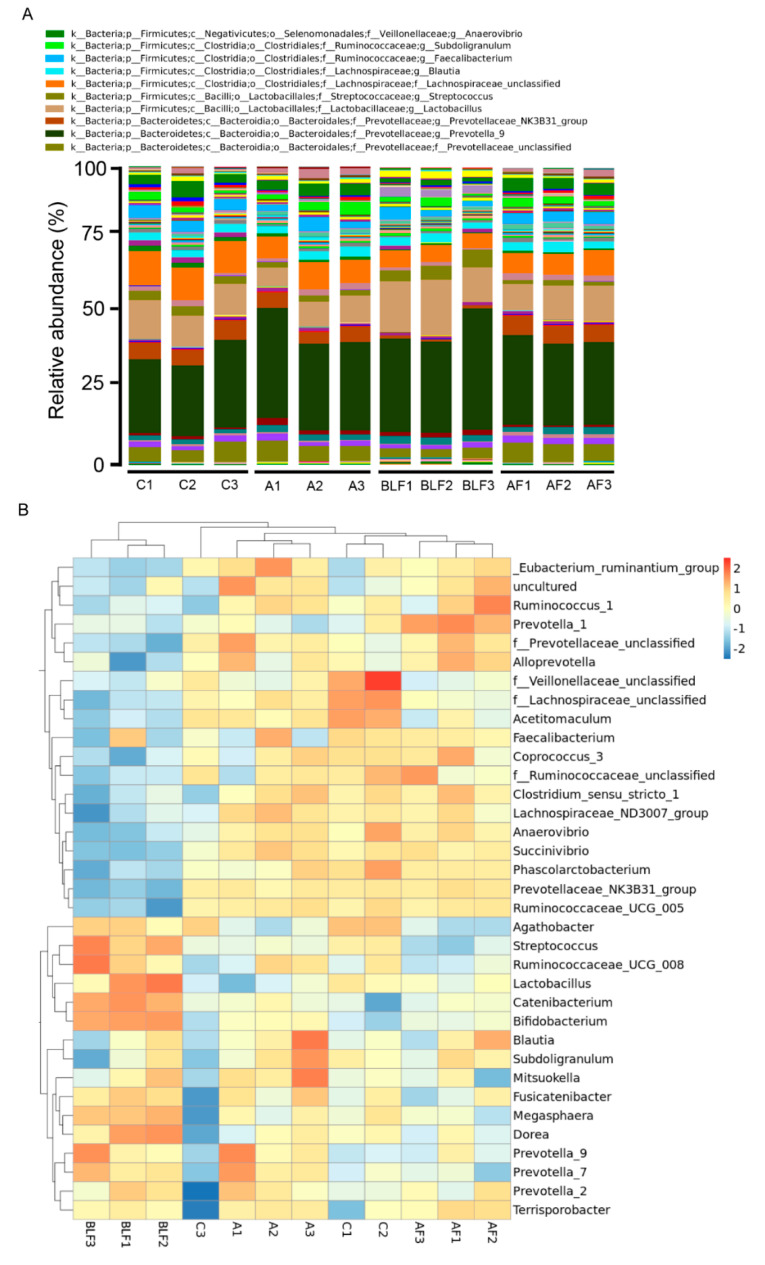
Taxonomic composition analysis of cecal digesta. (**A**) The microbiota compositions at the genus level for 16S rRNA sequences in cecal digesta. Composition of the major taxonomic groups at the genus level in samples collected from the C, A, BLF, and AF groups (n = 3). (**B**) Species abundance heat map showing the abundance distribution of the dominant 35 genera (*Y*-axis) among bacterial communities between groups (*X*-axis) (n = 3). Values are normalized by Z-score.

The results of bacterial taxonomy in the cecal digesta of weaning piglets are shown in Table 5. At the phylum level, no significant differences were found in the abundance of the Firmicutes and Bacteroidetes phyla between groups. Replacing all the bacitracin with BLF decreased the abundance of the Actinobacteria phylum compared with groups treated with antibiotics alone (*p* < 0.05). The Proteobacteria phylum abundances were increased in the group treated with BLF alone compared with other groups (*p* < 0.001). Replacing all the bacitracin with BLF increased the abundances of the Bacilli, Erysipelotrichia, and Coriobacteriia classes compared with other groups (*p* < 0.05). The Gammaproteobacteria class abundances were lower in the groups treated with BLF alone compared with the group treated with bacitracin alone (*p* < 0.05). The Aeromonadales order abundances were lower in the groups treated with BLF alone compared with the group treated with bacitracin alone (*p* < 0.05). Replacing all the bacitracin with BLF increased the abundance of the Lactobacillales, Erysipelotrichales, and Coriobacteriales orders compared with other groups (*p* < 0.05). The Lachnospiraceae family abundances were lower in all treatment groups compared with the control group (*p* < 0.05). The abundances of the Streptococcaceae and Erysipelotrichaceae families were increased in the groups treated with BLF alone compared with other groups (*p* < 0.05). Replacing all the bacitracin with BLF reduced the abundances of the Succinivibrionaceae and Muribaculaceae families compared with the group treated with BLF in combination with bacitracin (*p* < 0.05). The Peptostreptococcaceae family abundances were higher in all treatment groups compared with the control group (*p* < 0.05). Replacing all the bacitracin or half the bacitracin with BLF decreased the abundance of the *Lachnospiraceae_unclassified* genus compared with the control group (*p* < 0.05). The half replacement of bacitracin with BLF increased the abundance of the *Prevotellaceae NK3B31 group* genus compared with the group treated with bacitracin alone, while replacing all the bacitracin with BLF decreased the abundance of the *Prevotellaceae NK3B31 group* genus (*p* < 0.001). Replacing all the bacitracin with BLF increased the abundance of the *Streptococcus* genus compared with other groups (*p* < 0.05). Compared with the control group, the *Agathobacter* genus abundances were reduced in the group treated with bacitracin and the group treated with BLF in combination with bacitracin (*p* < 0.05). Replacing all the bacitracin with BLF decreased the abundance of the *Acetitomaculum* genus compared with the control group and the group treated with bacitracin alone (*p* < 0.05). An overview of the taxonomy at the genus level and a heat map of the 35 most abundant genera in the cecal digesta are shown in Figure 3. The results of the heat map showed that similar bacterial community clusters, such as the *Anaerovibrio*, *Succinivibrio*, *Phascolarctobacterium*, *Prevotellaceae NK3B31 group*, and *Ruminococcaceae_UCG_005* genera were observed between the groups treated with basal diet, bacitracin alone, and BLF in combination with bacitracin. In contrast, replacing all bacitracin with BLF resulted in unique bacterial community clusters compared with other groups, such as the *Streptococcus*, *Ruminococcaceae_UCG_008*, *Lactobacillus*, *Catenibacterium*, and *Bifidobacterium* genera. In addition, some bacterial community clusters were decreased in the groups treated with BLF alone, such as the *Anaerovibrio*, *Succinivibrio*, *Phascolarctobacterium*, *Prevotellaceae NK3B31 group*, and *Ruminococcaceae_UCG_005* genera. Some overlaps in bacterial community clusters were observed between the groups treated with bacitracin alone and the groups treated with BLF alone, such as the *Fusicatenibacter*, *Prevotella_9*, *Prevotella_7*, *Prevotella_2*, and *Terrisporobacter* genera.

### 3.4. Association between the Average Abundance of the Genera, Diarrhea Incidence, and Growth Performance

**Figure 4:**
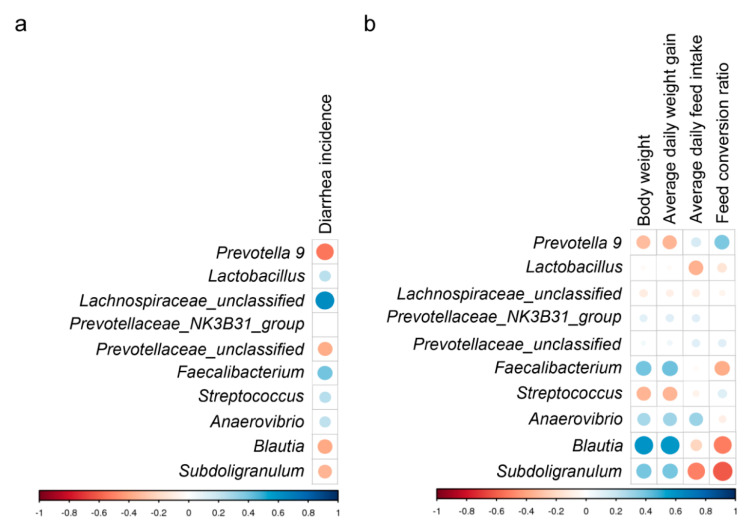
The correlation analysis between cecal microbiota, diarrhea incidence, and growth performance in weaning piglets. (**a**) Relationship between diarrhea incidence and abundant genera. (**b**) Relationship between growth performance and abundant genera. The size and shade of each circle represents the magnitude of correlation. The blue circle and red circle represent positive and negative correlations, respectively.

The *Prevotella 9*, *Prevotellaceae_unclassified*, *Blautia*, and *Subdoligranulu*m genera abundances were negatively associated with the incidence of diarrhea, whereas the *Lactobacillus*, *Lachnospiraceae_unclassified*, *Faecalibacterium*, *Streptococcu*s, and Anaerovibrio genera abundances were positively associated with the incidence of diarrhea (Figure 4a). The *Prevotella 9* and *Streptococcus* genera abundances were negatively associated with body weight (BW) and the average daily weight gain (ADG). In contrast, the *Faecalibacterium*, *Anaerovibrio*, *Blautia*, and *Subdoligranulum* genera abundances were positively correlated with body weight and average daily weight gain. In addition, the *Faecalibacterium*, *Blautia*, and *Subdoligranulum* genera abundances were negatively correlated with the feed conversion ratio (FCR), whereas the *Prevotella 9* genus abundances were positively correlated with the FCR (Figure 4b).

## 4. Discussion

In this study, we demonstrated for the first time that the full or half replacement of bacitracin with BLF reduced the incidence of diarrhea in weaning piglets. The principal coordinates analysis of qualitative traits and a heat map of species abundance revealed distinct clusters between the groups treated with bacitracin alone, BLF alone, and bacitracin in combination with BLF. The *Prevotellaceae NK3B31 group*, *Prevotellaceae_unclassified*, and *Anaerovibrio* genera abundances were higher in the cecal digesta of the group treated with bacitracin in combination with BLF. The *Prevotella 9*, *Prevotellaceae_unclassified*, *Blautia*, and *Subdoligranulum* genera abundances in the cecal digesta were negatively correlated with the diarrhea incidence of weaning piglets.

Previous studies have generally focused on the effects of multi-strains of *Bacillus* species-based direct-fed microbes on the growth performance and diarrhea incidence in weaning piglets [15,17,22]. The combined use of *B. licheniformis* and *B. subtilis* in weaning piglets was previously found to ameliorate daily weight gain and the feed conversion ratio, as well as to reduce the morbidity and mortality rates associated with diarrhea [15]. The dietary supplementation of *Bacillus* species-based probiotics (*B. lichenformis*, *B. subtilis*, and *Bacillus coagulans*) improved the average daily weight gain and feed efficiency in growing–finishing pigs [22]. Furthermore, the dietary supplementation of a combined mixture of *B. lichenformis* and *Clostridium butyricum* decreased the diarrhea incidence in weaning piglets [17]. Supplementing a BLF in the diet of weaning piglets alleviated the diarrhea incidence [11]. In the present study, we demonstrated that, similar to the effects of antibiotics, replacing all the bacitracin with BLF still reduced diarrhea incidence in weaning piglets. The half replacement of bacitracin with BLF not only alleviated the diarrhea incidence but also had a tendency to increase the body weight and average daily weight gain of weaning piglets. The live or spore form of *B. licheniformis* is the most widely used formula of probiotics for the prevention of post-weaning diarrhea in the diet of weaning piglets [15,17,22,23]. *B. licheniformis*-derived metabolites, such as antimicrobial peptides, are generally not included in the diet of weaning piglets. Here, the fermented feed additive contained the live *B. licheniformis* and *B. licheniformis*-derived antimicrobial peptide surfactin. It has been reported that surfactin isolated from BLF can inhibit the growth of diarrhea-associated pathogens, such as *Clostridium perfringens* and *Brachyspira hyodysenteriae* [14,24]. Surfactin also exhibited an anti-inflammatory effect without cytotoxicity in vitro [25,26]. In addition to *B. licheniformis*-derived surfactin, *B. licheniformis* reduced the pathogens in the intestine by competitive exclusion and increased nutrient utilization [12,27]. The antibiotic used in the present study, bacitracin, can be produced by *B. licheniformis* and has potent antibacterial activity, primarily against Gram-positive organisms [28,29]. Therefore, compared with previous studies, the mechanism or efficiency of BLF on the improvement of daily weight gain and the alleviation of diarrhea incidence in weaning piglets may be different, as the half replacement of bacitracin with BLF resulted in a superior average daily weight gain in weaning piglets compared with the groups treated with bacitracin or BLF alone. *B. licheniformis* and surfactin in the fermented products is likely to still be active in the gastrointestinal tract when simultaneously supplemented with bacitracin. Taken together, these findings demonstrate that the dietary supplementation of BLF has beneficial effects on the prevention of post-weaning diarrhea in piglets. The effects of replacement of antibiotics with different concentrations of BLF on the growth performance and diarrhea incidence in weaning piglets remain to be investigated in the future. In addition, the effects of a half dose of antibiotic treatment alone, a half dose of BLF treatment alone, and a half dose of antibiotics in combination with a half dose of BLF treatment on weaning piglets still need to be confirmed.

The intestinal microbiota are also critical in providing resistance against pathogen colonization [30]. Pathogen invasion and environmental stress are associated with post-weaning diarrhea by altering the intestinal microbiota composition [31]. Antibiotics are known to reduce the incidence of post-weaning diarrhea by destroying pathogenic bacteria and changing the gut microbial composition in piglets [1]. Imbalanced fecal microbiota have been observed in response to antibiotic prophylaxis in poultry and pigs [11,32]. In addition to antibiotics, gut microbial composition and diversity can be shaped by probiotics [32]. Many countries have prohibited the use of antibiotic growth promoters as feed additives, but livestock has become more prone to infection by pathogens, thereby negatively affecting production. Therefore, it is imperative to explore effective alternatives to antibiotic growth promoters in infectious disease prevention in animal production. The effect of replacing or reducing antibiotics with probiotics on the performance and gut microbiota of pigs has been increasingly investigated [18,19,23,33]. It has been reported that the simultaneous supplementation of *B. licheniformis* and antibiotics cannot improve the growth performance of weaning piglets [23]. Body weight and gut microbiota have not been found to be affected by the supplementation of *Bacillus* species-based probiotics in combination with antibiotic administration in piglets during lactation and post-weaning [33]. Replacing all the antibiotics or the half replacement of antibiotics with *B. amyloliquefaciens* has been found to increase growth performance, antioxidant capacity, and digestive enzyme activity compared with the groups treated with antibiotics alone [18,19]. However, the half and total replacement of antibiotics with *B. amyloliquefaciens* was found to not alter the α-diversity of the microbial population in the feces of weaning piglets [19]. However, a recent study demonstrated that bacterial species richness and evenness in the feces decreased in weaning piglets fed BLF or antibiotics [11]. In the present study, we demonstrated that no difference was found in the bacterial species richness in the cecal digesta between groups. These findings imply that the BLF or bacitracin differently regulate the microbiota between cecal digesta and fecal content in weaning piglets. Replacing all the bacitracin with BLF reduced the bacterial species evenness compared with the control group, while the effects were reversed in the group treated with BLF in combination with bacitracin. Mirroring the results of a previous study [11], a principal coordinate analysis with quantitative traits showed that bacterial communities were clearly distinct in the cecal digesta for groups treated with bacitracin alone, BLF alone, or both the bacitracin and the BLF. These findings demonstrated that the full of half replacement of bacitracin with BLF can have a different impact on the cecal microbiota of weaning piglets.

The gut commensal *Prevotella* species mainly contribute to polysaccharide breakdown and short-chain fatty acid production in the gut of weaning piglets [34,35]. The *Prevotella 9* genus is one of the dominant species in the gut and is associated with diarrhea incidence in weaning piglets. It has been demonstrated that the supplementation of fiber in the diet of piglets decreased the diarrhea incidence and increased the *Prevotella 9* genus abundances [35]. The *Prevotella 9* genus abundances in the fecal microbiota of diarrheic piglets were lower than those in non-diarrheic piglets [36]. The *Prevotella 9* genus abundances in the feces are negatively associated with the diarrhea incidence in weaning piglets, and supplementation with BLF has been shown to be able to increase the abundance of the *Prevotella 9* genus in the feces [11]. In the current study, the *Prevotella 9* genus abundances in the cecal digesta were also negatively associated with the diarrhea incidence of weaning piglets. Since the highest abundance of the *Prevotella 9* genus in the cecal digesta was not observed in the piglets with the superior growth performance (BLF in combination with the bacitracin-treated group), the *Prevotella 9* genus abundances in the cecal digesta were not positively correlated with the growth performance of weaning piglets, indicating that the *Prevotella 9* genus cannot be used as an indicator of growth performance in weaning piglets. Alternatively, the *Blautia* and *Subdoligranulum* genera in the cecal digesta may be potential indicators of growth performance and diarrhea incidence in weaning piglets. Previous studies have reported that the *Blautia* genus, a short-chain fatty acid-producing bacteria, is positively correlated with growth performance and negatively correlated with diarrhea incidence in weaning piglets [11,37]. The *Subdoligranulum* genus is known to be involved in the conversion of lactate to butyrate and has been positively associated with feed efficiency in pigs [38,39]. Taken together, the abundance of the *Prevotella 9* genus in the gastrointestinal tract plays a critical role in the prevention of diarrhea incidence in weaning piglets. However, how these bacteria interact with each other in response to bacitracin or BLF or both to alleviate diarrhea in weaning piglets remains to be elucidated. Since the pH, short-chain fatty acid concentration, and pro-oxidative and anti-oxidative balance of the cecal digesta are associated with gut microbiota, whether the changes of microbiota in response to antibiotics or BLF or both in the cecal digesta of weaning piglets also regulate these parameters remain to be investigated.

## 5. Conclusions

These findings confirmed that the half replacement of bacitracin with BLF has beneficial effects on the reduction of diarrhea incidence in weaning piglets. Similar to bacitracin, the full or half replacement of bacitracin with BLF can reduce diarrhea incidence. Furthermore, BLF and bacitracin differentially modulate the cecal microbiota of weaning piglets. Therefore, based on our research, a *Bacillus licheniformis*-fermented feed additive could be a feasible alternative to antibiotics in the piglet diet for the reduction of antibiotic use.

## Figures and Tables

**Table 1:** Composition of basal diets.

Item	d 1 to 28 ^1^
Ingredient, g kg^−1^	
Corn, yellow	570.0
Soybean meal, 44% crude protein (CP)	260.0
Dried whey	85.0
Soybean oil	35.0
Fish meal, 60% CP	30.0
CaHPO_4_	12.0
CaCO_3_, 38% Ca	5.0
Salt	4.0
L-Lysine, 98%	3.5
Vitamin, premix ^2^	1.0
Mineral, premix ^3^	1.0
Choline, 50%	0.8
Fermented feed additive	0
Chemical composition, g kg^−1^	
Crude protein	191.2
Phenylalanine and Tyrosine	15.5
Lysine	14.5
Methionine and Cystine	6.5
Analyzed calcium	8.0
Analyzed total phosphorus	7.0
Metabolizable energy (ME), kcal/kg	3567.63

^1^ Fed in meal form to all piglets; ^2^ supplied per kg diet: vitamin A, 6000 international unit (IU); vitamin D, 900 IU; vitamin E, 30 IU; vitamin K3, 3 mg; vitamin B2, 6 mg; pantothenic acid, 18 mg; niacin, 60 mg; and vitamin B12, 30 μg; ^3^ supplied per kg diet: Fe, 140 mg; Zn, 100 mg; Cu, 20 mg; Mn, 4 mg; I, 0.2 mg; and Se, 0.1 mg.

**Table 2:** Effect of bacitracin and *Bacillus licheniformis*-fermented feed additive (BLF) on the diarrhea incidence of weaning piglets.

Item	C ^1^	A ^2^	BLF ^3^	AF ^4^	SEM ^5^	*p*-Value
Diarrhea, %						
1–14 d	16.06 ^6,a^	6.84 ^b^	10.11 ^b^	9.22 ^b^	1.264	0.009
15–28 d	11.26	8.11	8.41	6.59	0.855	0.373
1–28 d	13.66	7.48	9.27	7.91	0.968	0.058

^1^ C = Basal diet; ^2^ A = basal diet supplemented with 30 mg/kg bacitracin; ^3^ BLF = basal diet supplemented with 1 g/kg BLF; ^4^ AF = basal diet supplemented with 15 mg/kg bacitracin and 0.5 g/kg BLF; ^5^ SEM = standard error of mean; ^6^ data are mean values of 3 replicate per treatment (8 piglets per replicate); ^a,b^ means that have no superscript in common are significantly different from each other (*p* < 0.05).

**Table 3:** Effects of bacitracin and BLF on small intestine morphology.

Item		C ^1^	A ^2^	BLF ^3^	AF ^4^	SEM ^5^	*p*-Value
Villus length (μm)	Duodenum	519.35 ^6,b^	668.85 ^a^	597.35 ^ab^	611.00 ^ab^	26.546	0.008
	Jejunum	401.70	480.35	425.75	466.70	13.067	0.251
	Ileum	260.49	536.25	635.05	490.75	52.362	0.199
Crypt depth (μm)	Duodenum	156.00	134.55	139.75	107.25	9.851	0.818
	Jejunum	170.95	107.25	97.5	102.05	11.641	0.062
	Ileum	114.40	117.00	146.25	160.55	10.841	0.743
Villus length: Crypt depth	Duodenum	3.44	5.32	4.28	5.81	0.374	0.528
	Jejunum	2.38	4.89	4.71	4.59	0.424	0.138
	Ileum	2.34 ^b^	4.69 ^a^	4.39 ^a^	3.08 ^ab^	0.324	0.035

^1^ C = Basal diet; ^2^ A = basal diet supplemented with 30 mg/kg bacitracin; ^3^ BLF = basal diet supplemented with 1 g/kg BLF; ^4^ AF = basal diet supplemented with 15 mg/kg bacitracin and 0.5 g/kg BLF; ^5^ SEM = standard error of mean; ^6^ data are mean values of 3 replicate per treatment (2 piglets per replicate); ^a,b^ means that have no superscript in common are significantly different from each other (*p* < 0.05).

**Table 4:** The microbial alpha diversity in the cecal digesta of weaning piglets. OTU: operational taxonomic unit.

Item	Effective Reads	Number of OTUs	Chao1	Fisher Alpha	Shannon	Simpson Reciprocal
C ^1^	84,566.00 ^7^	20,138.67	1352.67	250.88	7.62 ^a^	48.81 ^ab^
A ^2^	97,807.33	23,335.67	1105.00	189.72	7.41 ^a^	39.44 ^bc^
BLF ^3^	87,001.33	21,042.00	951.67	160.89	6.98 ^b^	36.59 ^c^
AF ^4^	89,312.00	21,133.00	1156.67	204.37	7.64 ^a^	50.96 ^a^
SEM ^5^	2244.360	514.455	53.385	12.787	0.083	2.189
Adjusted *p*-Value ^6^	0.167	0.167	0.172	0.202	0.006	0.054

^1^ C = Basal diet; ^2^ A = basal diet supplemented with 30 mg/kg bacitracin; ^3^ BLF = basal diet supplemented with 1 g/kg BLF; ^4^ AF = basal diet supplemented with 15 mg/kg bacitracin and 0.5 g/kg BLF; ^5^ SEM = standard error of mean; ^6^ adjusted *p*-value: false discovery rate correction using the Benjamini–Hochberg procedure; ^7^ data are mean values of 3 replicate per treatment (2 piglets per replicate); ^a–c^ means that have no superscript in common are significantly different from each other (*p* < 0.05).

**Table 5:** Comparison of the abundances of bacterial taxa in the cecal digesta of weaning piglets.

Item	Relative Abundance (%) ^1^					Adjusted
	C ^2^	A ^3^	BLF ^4^	AF ^5^	SEM ^6^	*p*-Value ^7^
Phylum						
Firmicutes	54.30 ^8^	45.79	51.99	48.05	1.655	0.379
Bacteroidetes	43.46	50.24	45.50	49.12	1.630	0.468
Actinobacteria	1.43 ^ab^	2.84 ^a^	0.36 ^b^	1.88 ^ab^	0.309	0.018
Proteobacteria	0.47 ^c^	0.82 ^b^	2.05 ^a^	0.67 ^bc^	0.188	<0.001
Class						
Bacteroidia	43.46	50.23	45.50	49.12	1.630	0.468
Clostridia	31.90	27.92	22.21	27.86	1.279	0.059
Bacilli	14.20 ^b^	9.91 ^b^	20.67 ^a^	12.18 ^b^	1.315	0.012
Negativicutes	7.64	7.19	5.45	7.12	0.465	0.468
Gammaproteobacteria	1.36 ^ab^	2.75 ^a^	0.34 ^b^	1.81 ^ab^	0.302	0.018
Erysipelotrichia	0.54 ^b^	0.77 ^b^	3.61 ^a^	0.86 ^b^	0.387	<0.001
Coriobacteriia	0.21 ^b^	0.17 ^b^	0.87 ^a^	0.25 ^b^	0.089	<0.001
Order						
Bacteroidales	43.35	50.09	45.44	49.04	1.625	0.469
Clostridiales	31.90	27.92	22.21	27.86	1.279	0.059
Lactobacillales	14.20 ^b^	9.90 ^b^	20.67 ^a^	12.18 ^b^	1.316	0.012
Selenomonadales	7.64	7.19	5.45	7.12	0.465	0.469
Aeromonadales	1.08 ^ab^	2.23 ^a^	0.09 ^b^	1.63 ^a^	0.276	0.025
Erysipelotrichales	0.54 ^b^	0.77 ^b^	3.61 ^a^	0.86 ^b^	0.387	<0.001
Coriobacteriales	0.21 ^b^	0.17 ^b^	0.87 ^a^	0.25 ^b^	0.088	<0.001
Family						
Prevotellaceae	42.59	49.70	45.20	48.49	1.645	0.490
Lachnospiraceae	21.10 ^a^	16.30 ^b^	13.66 ^b^	15.43 ^b^	0.876	0.003
Lactobacillaceae	11.38	7.96	15.79	10.65	1.012	0.074
Ruminococcaceae	8.48	8.41	6.44	8.88	0.475	0.406
Veillonellaceae	6.66	6.52	5.22	6.33	0.380	0.618
Streptococcaceae	2.82 ^b^	1.94 ^b^	4.88 ^a^	1.53 ^b^	0.414	0.003
Clostridiaceae_1	1.47	1.78	0.86	1.90	0.156	0.109
Acidaminococcaceae	0.97	0.67	0.22	0.79	0.103	0.093
Succinivibrionaceae	1.08 ^ab^	2.23 ^a^	0.09 ^b^	1.63 ^a^	0.276	0.028
Erysipelotrichaceae	0.54 ^b^	0.77 ^b^	3.61 ^a^	0.86 ^b^	0.387	<0.001
Muribaculaceae	0.39 ^a^	0.26 ^bc^	0.19 ^c^	0.31 ^ab^	0.024	<0.001
Peptostreptococcaceae	0.52 ^b^	1.21 ^a^	1.13 ^a^	1.39 ^a^	0.114	0.019
Genus						
*Prevotella 9*	25.97	32.05	34.31	28.47	1.380	0.179
*Lactobacillus*	11.38	7.96	15.79	10.65	1.012	0.081
*Lachnospiraceae_unclassified*	11.06 ^a^	8.09 ^b^	5.45 ^c^	7.51 ^bc^	0.639	0.004
*Prevotellaceae NK3B31 group*	5.71 ^ab^	4.76 ^b^	1.00 ^c^	6.16 ^a^	0.624	<0.001
*Prevotellaceae_unclassified*	5.18	5.74	3.24	6.12	0.417	0.081
*Faecalibacterium*	3.80	2.88	2.76	3.65	0.286	0.644
*Streptococcus*	2.82 ^b^	1.94 ^b^	4.88 ^a^	1.53 ^b^	0.414	0.004
*Anaerovibrio*	3.64 ^ab^	3.65 ^ab^	1.46 ^b^	3.87 ^a^	0.360	0.080
*Blautia*	2.55	2.95	2.68	2.94	0.133	0.720
*Subdoligranulum*	1.71	2.78	1.68	2.28	0.275	0.644
*Agathobacter*	1.80 ^a^	0.01 ^b^	0.83 ^ab^	0.01 ^b^	0.238	0.006
*Alloprevotella*	1.81	1.82	1.36	2.19	0.114	0.108
*Prevotella 2*	1.56	2.09	2.25	2.08	0.114	0.179
*Acetitomaculum*	1.48 ^a^	0.89 ^b^	0.40 ^c^	0.63 ^bc^	0.127	<0.001
*Clostridium_sensu_stricto_1*	1.46	1.78	0.86	1.90	0.156	0.123

^1^ Bacterial taxa present at relative abundances of greater than 0.5% and the proportion of unidentified bacteria is 0.022%; ^2^ C = basal diet; ^3^ A = basal diet supplemented with 30 mg/kg bacitracin; ^4^ BLF = basal diet supplemented with 1 g/kg BLF; ^5^ AF = basal diet supplemented with 15 mg/kg bacitracin and 0.5 g/kg BLF; ^6^ SEM = standard error of mean; ^7^ adjusted *p* value: false discovery rate correction using the Benjamini–Hochberg procedure; ^8^ data are mean values of 3 replicate per treatment (2 piglets per replicate); ^a–c^ means that have no superscript in common are significantly different from each other (*p* < 0.05).
